# TAFFEL: Independent Enrichment Analysis of gene sets

**DOI:** 10.1186/1471-2105-12-171

**Published:** 2011-05-19

**Authors:** Mitja I Kurki, Jussi Paananen, Markus Storvik, Seppo Ylä-Herttuala, Juha E Jääskeläinen, Mikael von und zu Fraunberg, Garry Wong, Petri Pehkonen

**Affiliations:** 1 Laboratory of Functional Genomics and Bioinformatics, Department of Neurobiology, A.I. Virtanen Institute for Molecular Sciences, University of Eastern Finland, PO Box 1627, FIN-70211 Kuopio, Finland; 2 Department of Biosciences, University of Eastern Finland, PO Box 1627, FIN-70211 Kuopio, Finland; 3 Department of Pharmacology and Toxicology, University of Eastern Finland, PO Box 1627, FIN-70211 Kuopio, Finland; 4 Department of Biotechnology and Molecular Medicine, A.I. Virtanen Institute for Molecular Sciences, University of Eastern Finland, PO Box 1627, FIN-70211 Kuopio, Finland; 5 Department of Neurosurgery, Kuopio University Hospital, FIN-70211 Kuopio, Finland; 6 Institute of Clinical Medicine, University of Eastern Finland, PO Box 1627, FIN-70211 Kuopio, Finland

## Abstract

**Background:**

A major challenge in genomic research is identifying significant biological processes and generating new hypotheses from large gene sets. Gene sets often consist of multiple separate biological pathways, controlled by distinct regulatory mechanisms. Many of these pathways and the associated regulatory mechanisms might be obscured by a large number of other significant processes and thus not identified as significant by standard gene set enrichment analysis tools.

**Results:**

We present a novel method called Independent Enrichment Analysis (IEA) and software TAFFEL that eases the task by clustering genes to subgroups using Gene Ontology categories and transcription regulators. IEA indicates transcriptional regulators putatively controlling biological functions in studied condition.

**Conclusions:**

We demonstrate that the developed method and TAFFEL tool give new insight to the analysis of differentially expressed genes and can generate novel hypotheses. Our comparison to other popular methods showed that the IEA method implemented in TAFFEL can find important biological phenomena, which are not reported by other methods.

## Background

Gene expression studies often compare samples from two or more experimental conditions, the most typical outcome being a set of genes that differ in expression between the conditions. Several databases, computational methods and software programs have been recently published for analysis of such differentially expressed (DE) gene sets. Usually these tools are aimed at finding out associated (differentially active) biological mechanisms by searching associations of DE genes to various biological functions, processes and pathways reported in the biological databases such as Gene Ontology (GO) [1]. The output of these tools is usually a list of biological terms (functions, processes, pathways etc.) that are more frequently associated to the gene set than expected by chance. Therefore, this analysis is often referred to as enrichment analysis (EA) (for an extensive review of these methods see [2]). This type of analysis is implemented in tools such as GENERATOR [3], DAVID [4], FatiGO [5], GOToolBox [6], GenMAPP [7], GoMiner [8], Gostat [9] and OntoTools [10].

Standard EA has some notable shortcomings that should be taken into account, especially in the case of DE genes. First, DE genes tend to be associated to multiple distinct biological phenomena rather than one or a few. This problem has been recently addressed by applying various clustering methods for finding gene subgroups with homogeneous functional annotations [3,4,6], and combining similar functional annotations together [4]. Clustering can reveal interesting gene subgroups, but so far, there are no definitive methods available to verify them or obtain further interpretation about their biological significance in the studied cases, other than calculating the internal homogeneity of clusters. Secondly, the result of EA is largely dependent on statistical cut-off values used for selecting the list of DE genes. By choosing a loose cut-off value, many important processes may be obscured by false positive (FP) genes and thus are not observed. This is partly addressed by the aforementioned clustering methods, which can separate FP genes [3] and methods like Gene Set Enrichment Analysis (GSEA) [11] and Functional Class Scoring (FCS) [12], not based on fixed cut-off. Still, these methods do not show any further evidence about the importance of resulting genes or gene groups.

**Figure 1:**
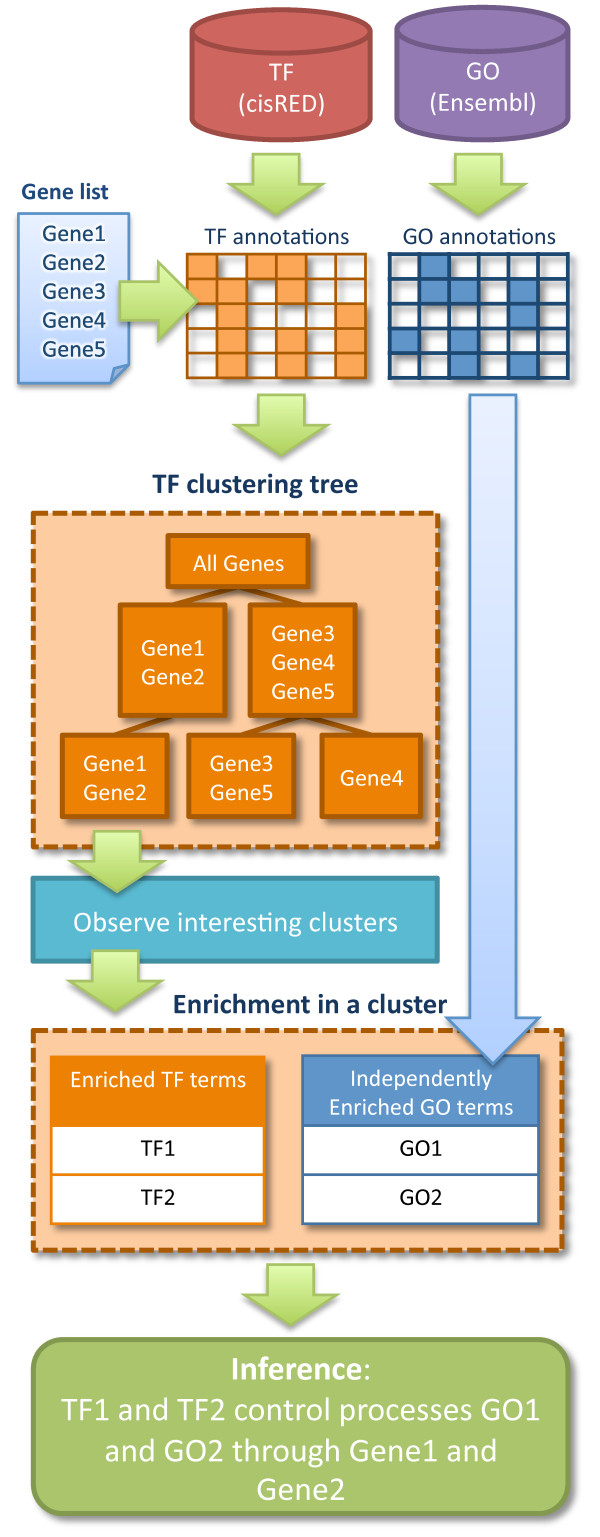
**The flow diagram of TAFFEL analysis**. From the top: the list of genes given by the user is annotated by GO and TF information from Ensembl (20) and cisRED (12) databases. The genes are clustered separately in parallel, based on GO and TF annotations (for simplicity only the TF clustering tree is shown). In each resulting cluster, the enrichment of both GO and TF annotations is determined, providing a basis for suggesting implications between the biological processes and their regulator molecules.

In this article, we present a novel method Independent Enrichment Analysis (IEA) and its implementation in a software tool called TAFFEL. The principal idea of IEA and TAFFEL is to facilitate the discovery of relevant biological phenomena from a set of differentially expressed genes and potential mechanisms of the regulation of those processes. The developed application allows quick and easy explorative analysis of data by performing three main steps (Figure 1). First, TAFFEL uses functional annotations from Gene Ontology [1] to separate differentially expressed genes into functionally homogenous gene groups. This facilitates the discovery of multiple biological phenomena associated to DE genes. Secondly, TAFFEL discovers groups of genes with similar *cis*-regulatory transcription factor binding sites (TFBSs) in their regulatory regions, using annotations of TFBS to specific transcription factors (TF) from cisRED database [13]. This enables finding putatively co-regulated genes from the gene list and identification of their regulators. At this point, the analyst has several groups of genes that are homogenous in either GO or TF annotations. Therefore, as a third step TAFFEL includes a novel method referred to as Independent Enrichment Analysis (IEA) which evaluates the enrichment of TFs in gene clusters homogeneous in GO terms, and vice versa, enrichment of GO terms in gene clusters homogeneous in TF annotations. IEA provides clues to the regulatory control of genes sharing common functions. Simultaneously, it serves as an extrinsic biological validation of the obtained gene groups that can be used to point out the most interesting gene clusters among several. A detailed description of typical analysis flow with TAFFEL is provided in *Methods *and drawn in Figure 1.

In order to demonstrate the utility of our method and the associated software, we applied TAFFEL to two datasets. Firstly, we analyzed differentially expressed genes in human HEK293T cell culture after treatment with forskolin, a cyclic AMP (cAMP) pathway inducer. Using TAFFEL we show that the list of differentially expressed genes comprise separate functional and regulatory gene subsets that relate to parts of cAMP related pathways. The result indicates correctly that there are also other major mechanisms launched by cAMP besides the CREB binding protein related pathway that is most commonly linked to cAMP in the literature.

Secondly, we analyzed differentially expressed genes between human ruptured and unruptured saccular intracranial artery aneurysm (sIA) walls obtained during surgery. Subarachnoid hemorrhage from ruptured sIA (aSAH) is a devastating form of stroke that affects working age population [14]. The sIA disease is a complex trait that is poorly understood. In previous comparisons of ruptured and unruptured sIA walls, intimal hyperplasia, endothelial injury, luminal thrombosis, mitosis, apoptosis, T-cell and macrophage infiltration [15], expression of growth factor receptors [16], complement activation [17] and MAPK-signalling [18] were associated with the sIA wall rupture. In addition, in our whole genome mRNA profiling of 11 ruptured and 8 unruptured sIA walls inflammation, response to turbulent blood flow, leukocyte migration, oxidative stress and vascular remodelling were associated to the rupture and *In Silico *transcription factor analyses identified enriched NF-κB, HIF1A and ETS transcription factor binding sites among up-regulated genes [19]. This dataset was re-analyzed using TAFFEL in order to demonstrate the capability of TAFFEL to find novel phenomena overlooked in standard analysis and to identify factors that might be causing the reported phenomena. The results suggest novel molecular mechanisms and demonstrate the usefulness of TAFFEL in snapshot type research settings and in diseases of poorly characterized molecular pathogenesis.

We compared TAFFEL gene clustering results against results from five other methods or tools used for enrichment analysis: standard list of GO-terms sorted according to Fisher's Exact test p-values, a sorted list of GO-terms and transcription factors resulting from FatiGO+ tool [5], annotation sets resulting from the Functional Annotation Clustering tool available in DAVID [4], co-occurring sets of GO-terms and transcription factors resulting from apriori association rule discovery algorithm implemented in GeneCodis [20] and results from GSEA [11]. The comparison shows that TAFFEL can discover important individual themes and relations between transcription factors and biological processes that are not reported at all by other methods.

## Results

### Description of the method and tool

TAFFEL uses a non-nested hierarchical clustering scheme [3] for finding gene subgroups that are homogeneous in GO terms or TF annotations. The gene subgroups are a partition of the whole gene set i.e. they are disjoint sets that cover the whole gene set.

**Figure 2:**
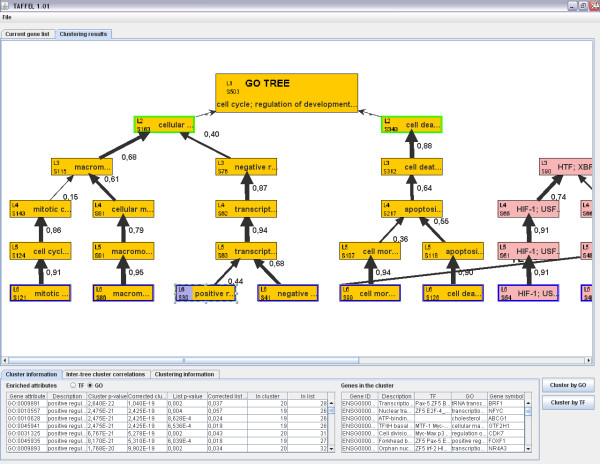
**TAFFEL user interface**. The clustering trees represents the clustering result for the DE genes after 4 hours of forskolin treatment in HEK293T cells. The genes have been clustered by the GO terms (left) and TFs (right). The topmost box represents the whole gene set without clustering. Below that, each level represents clustering to two, three, or more clusters. The green outline indicates the cluster number selection by AIC and blue by dAIC. The clusters obtained from the IEA analysis with FDR p < 0.1 are highlighted with the light blue background on the right side of cluster box. The best intercorrelating clusters (cell morphogenesis cluster in the GO tree and COUP cluster in the TF tree) between the trees are connected with the bold line. Information at bottom shows enriched annotations (left list) and cluster genes (right list). *Positive regulation of biosynthetic process *related cluster is selected in the picture.

The clustering of genes is performed using only GO or TF data and no gene expression data is needed. The method creates multiple clustering solutions with different numbers of clusters and combines them into a single visualization. Each clustering solution is visualized as a set of horizontally ordered rectangles, each rectangle representing a single cluster (Figure 2). Different clustering solutions are ordered vertically according to the number of clusters. Thus, the visualization contains several levels, the top level representing the whole gene list as a single cluster, the second level representing clustering of genes into two clusters, the third level representing solution with three clusters etc. The best correlating clusters between adjacent levels are combined with edges, creating a tree-like structure. Unlike in regular hierarchical clustering, the different tree levels are independent of each other. This visualization can be used to track coherent clusters that stay similar in different solutions despite the changing number of clusters and initialization for clustering, and to observe the hierarchical relationships in the data. In addition, the tool performs automatic evaluation of clustering solutions with different number of clusters using a statistical model selection (see Selection of number of clusters). The best scoring levels are highlighted in the visualization. The tree that is obtained using GO terms as data for clustering is referred to as "GO tree" and the tree obtained using TF annotations is referred to as "TF tree".

For each gene cluster, TAFFEL reports both the enriched GO terms and TF annotations, regardless of what information (GO or TF) was used for clustering. For the first level of the tree, representing the whole analyzed gene list, the enrichment is measured in the list versus the genome. This is analogous to the traditional enrichment analysis and can be used for observing the most interesting themes in general. This enrichment is also reported for the annotations in the clusters of subsequent tree levels (column "List p-value" in the software) as additional evidence of their biological significance. However, as a principal description for each cluster in the subsequent tree levels, TAFFEL reports annotations that are enriched in each cluster versus the whole gene list (column "Cluster p-value" in the software). This gives the user a compact overview of the different biological phenomena present in the analyzed list of genes.

In order to gain more evidence about the biological meaningfulness of resulting clusters, TAFFEL performs two types of extrinsic evaluation steps. Firstly, in the IEA evaluation, each functionally homogeneous gene cluster is evaluated in terms of enrichment of TFs, and each gene cluster homogeneous in TFs is evaluated in terms of enrichment in GO terms. Secondly, TAFFEL allows measuring correlations of gene memberships between all possible cluster pairs where one cluster comes from the GO tree and another from the TF tree. This measure, referred to as *inter-correlation*, can be used to identify the gene clusters that share same genes regardless of using TF's or GO terms as a basis for clustering. Both the IEA and inter-correlation can be used for validating the biological significance of gene subgroups, and to interpret relations between transcription regulators and processes they regulate.

### Availability and running the program

TAFFEL is a Java Web Start application written using Java Standard Edition 6 with NetBeans integrated development environment (http://www.netbeans.org). MySQL (http://www.mysql.com) database is used to store all the persistent data. Running TAFFEL requires Java Runtime Environment version 6. TAFFEL program, help-pages and example data sets are freely available under LGPL license from http://www.oppi.uku.fi/bioinformatics/taffel.

### Typical analysis flow

A typical analysis flow with TAFFEL is shown in Figure 1. Firstly, the gene list is imported to TAFFEL and clustered using GO terms and TF annotations as data. Secondly, the root levels of the GO and TF trees are observed to study the themes associated to the whole gene list in general. Thirdly, the clusters at the tree levels with the smallest dAIC scores in both the GO and TF trees are observed in order to find which separate themes are associated to the analyzed gene list and which respective gene subgroups constitute it. Fourthly, the coherency of these clusters is evaluated by observing their conservation throughout the tree. Finally, special focus is set on the clusters in the selected levels by using IEA and inter-correlation methods for cluster evaluation. The independently enriched themes in each cluster can be used to infer the TFs that drive a particular biological process or function in the analyzed condition.

The resulting clusters can be further analyzed by multiple ways such as highlighting the clusters including particular GO terms or TFs, to find correlations between clusters in different trees, and to show the list of genes associated to specific GO terms and/or TF annotations in each cluster. The results can be exported from the program in text form, and all results can be saved in one XML file.

### Analysis of forskolin effect on HEK293T cells

In order to test the developed method, we applied TAFFEL for the analysis of differentially expressed genes in HEK293T cells incubated with forskolin for four hours [21]. Forskolin increases the concentration of intracellular cyclic adenosine monophosphate (cAMP), a key mediator in several signalling pathways. The genes were clustered with TAFFEL using separately the GO *biological process *terms and TF annotations up to 15 clusters. The results were interpreted using the typical analysis flow described in *Methods*. Special attention was paid on the level at depth 11 in the GO tree and the level at depth 13 in the TF tree, both of which having obtained the best dAIC scores.

As expected, the results from enrichment of the complete gene list indicated that forskolin had overtaken the cAMP pathway from the G-protein receptor controlled pathways at the 4 h check point as there were a large number of genes induced by cAMP related GO terms. However, the TAFFEL clustering was able to detect a more complex network of interactions between the MAPK and AhR pathways. The gene clusters in dAIC selected level from the TF tree was enriched with certain expected TFs, such as ATF and CREB, AhR and HIF, variable E2 family and Rb complexes and EF dimers, and EGR-1. In turn, the clusters from the GO tree were enriched with lipid metabolism, cell adhesion, macromolecule localization, DNA metabolism and apoptosis related terms. Most of these clusters were also conserved at several tree levels, suggesting their coherency.

Two clusters in the GO tree included significantly enriched TFs when using IEA (see Table 1) one of them having two significantly enriched TFs. This cluster included 26 genes associated to transcription related themes and *macromolecule biosynthetic process *and related GO terms with HES-1 (FDR corrected p = 0.02) in 8 genes, and AhR (FDR corrected p = 0.029) in 15 genes enriched independently. This independent enrichment suggests looking further at the genes in this cluster. These were shown to include important regulators for proliferation. Another cluster found in IEA included 50 genes, and it was related to *macromolecule localization *and independently enriched the FOXO1 TF (see Table 1). There are several GTPase genes, with transport related functions, in this cluster many of which are reported to have binding site for FOXO1.

**Table 1:** Statistically significant clusters in the forskolin (FSK) and up-regulated (sIA↑) and down-regulated (sIA↓) aneurysm datasets

CLUSTER			ANNOTATION	P	P LIST	N	N LIST
FSK	GO	26	transcription from RNA polymerase II promoter	1.9E-21	8.2E-04	23	46
			positive regulation of macromolecule biosynthetic process	2.5E-21	5.7E-02	19	26
			positive regulation of gene expression	2.5E-21	2.4E-02	19	26
			HES-1	2.0E-02	7.2E-01	8	32
			AhR	2.9E-02	4.9E-02	15	123

FSK	GO	50	macromolecule localization	8.9E-37	8.2E-02	39	49
			protein transport	8.9E-37	1.0E-01	37	43
			establishment of protein localization	8.9E-37	1.1E-01	37	43
			FOXO1	3.3E-02	5.1E-01	10	32

FSK	TF	33	E2F-4_DP-2	4.2E-34	1.3E-01	31	48
			Rb_E2F-1_DP-1	4.2E-34	1.5E-01	30	43
			E2F-4_DP-1	6.2E-31	2.2E-02	29	45
			organelle organization	2.5E-03	4.0E-03	12	50

sIA ↑	GO	58	cation transport	3.8E-08	1.8E-01	17	23
			ion transport	3.8E-08	3.7E-01	18	26
			metal ion transport	1.2E-05	4.2E-01	12	16
			MTF-1	4.8E-02	4.7E-01	13	30
			ATF-1	4.8E-02	6.5E-01	9	17

sIA ↑	TF	29	S8	1.3E-19	8.5E-01	23	40
			Chx10	3.8E-14	7.3E-01	20	42
			Lhx3a	8.1E-13	6.7E-01	19	42
			amine biosynthetic process	9.1E-02	1.7E-01	3	4

sIA ↓	GO	22	nervous system development	1.3E-11	1.3E-02	17	32
			generation of neurons	2.1E-06	2.8E-01	8	10
			cell development	2.1E-06	2.8E-01	10	17
			Tal-1	3.1E-02	1.0E+00	4	5
			AR	9.4E-02	9.8E-01	6	17

sIA ↓	GO	49	organic acid metabolic process	1.3E-08	2.8E-01	13	13
			carboxylic acid metabolic process	1.3E-08	2.8E-01	13	13
			oxidation reduction	1.2E-07	2.8E-01	14	16
			lipid metabolic process	5.3E-07	4.4E-01	13	16
			NF-1	3.7E-02	9.8E-01	14	30

CLUSTER column indicates the clustered dataset, annotations used for clustering (either GO or TF) and the size of the cluster, respectively. ANNOTATION column indicates enriched GO terms and TF annotations from TRANSFAC in each cluster. P and P LIST columns indicate Benjamini-Hochberg FDR corrected Fisher's exact test p-values for the enrichment of the annotation in the cluster and in the gene list, respectively. N and N LIST columns show the number of genes associated with the annotation in the cluster and in the gene list.

The gene clusters were compared between GO and TF trees using *inter-correlation *method (data not shown). This analysis brought up again the GO cluster with *positive regulation of macromolecule biosynthetic process *as the highest correlating pair in the TF-tree being HIF-1A related gene cluster. As the HIF1A, one of the few hypoxia inducible factors, is the closest paralog to AhR in human, and both factors require ARNT or ARNT2 as dimerization partner, this even further suggests that the basic-helix-loop-helix transcription factors such as AhR have a role in cAMP signalling activated transcription.

### Analysis of activated and deactivated genes in ruptured intracranial aneurysm wall

We also applied TAFFEL to the analysis of differentially expressed genes in ruptured human sIA walls as compared to unruptured sIA walls. The overexpressed (marked with sIA↑) and the under expressed genes were clustered separately by using TF annotations and GO terms up to 20 clusters as this seemed to far exceed the best scoring cluster number according to dAIC measure. As this data set was already analyzed using standard enrichment method [19], we focused only on the IEA and *inter-correlation *methods in the best scoring clustering levels: level 8 for the GO tree and level 11 for the TF tree.

A few of these clusters obtained significant independent enrichment in IEA after correction for multiple testing (table 1). One was the protein phosphorylation (MAP kinases) and cation transport-related cluster among the over expressed genes. MAPK-signalling (MAPKS) in the sIA wall has previously been shown to be associated with rupture [18]. In IEA, MTF-1 (metal responsive transcription factor 1) was significantly independently enriched (FDR corrected p = 0.048). MTF-1 is stress and metal-activated (especially zinc) TF and drives the expression of antioxidant and anti-inflammatory genes, e.g., in atherosclerosis [22]. It controls, for example, metallothioneins (MT), zinc-transferring proteins. The cluster is enriched in ion-transferring proteins and contains MT2A, a primary target of MTF-1.

Another cluster found in IEA from the analysis of under expressed genes was related to oxidation-reduction and independently enriched the NF-1 (nuclear factor 1 C, NF1C) transcription factor (FDR corrected p = 0.037). NF1C activation capability is repressed by oxidative stress and NFIC knockout decreases the activity of Cytochrome p450 family gene CYP1A1 [23]. The cluster contains 2 CYP-family genes, and many other lipid and amino acid metabolizing genes as well as genes protecting against or controlling oxidative stress (NXN, OXR1).

The third cluster found in IEA was identified among the down-regulated genes. The cluster was enriched with neuron development related GO terms, *cell development*, *cell motion *(not visible among top three), *cell projection and organization *(not visible among top three) and independently enriched Tal-1 transcription factor (FDR corrected p = 0.031). Tal-1 protein is known to drive endothelial cell migration and morphogenesis in angiogenesis [24,25]. Tal-1 regulates VE-cadherin expression in endothelial cells. VE-cadherin concentrates on cell-to-cell adherens junctions and maintains cell adhesion, controls vascular permeability and relays signals necessary for vascular stabilization. VE-cadherin is a positive controller of TGF-β signalling and deletion of various components of this signalling pathway leads to several vascular manifestations, often including hemorrhages [26].

In order to find out whether the clustering by GO terms and TF annotations would yield any clusters with common genes, TAFFEL *inter-correlation *method was applied. The link between apoptosis and TFs MEF2A and Lhx3a was strongly observed (FDR corrected p = 7.5E-6). MEF2A is a myocyte enhancer factor, which controls many muscle-specific genes. Low number of smooth muscle cells with disorganized architecture has been associated with aneurysm rupture [15]. MEF2A has also been implicated as a candidate gene for coronary artery disease, and our results suggest that MEF2A dysregulation might be involved in smooth muscle cell apoptosis in the ruptured sIA walls.

### Comparison of TAFFEL to other methods

Several different approaches for analyzing differentially expressed gene sets exists, such as GENERATOR [3], DAVID [4], FatiGO [5], GOToolBox [6], GenMAPP [7], GoMiner [8], OntoTools [10], and GSEA [11], which can report the enriched terms e.g. the functional annotations, or TF information but no relation between these concepts. The main advancement of TAFFEL is that the developed IEA method, which allows statistically interpretable evaluations for the found clusters, helps to pay attention to the most interesting gene clusters among many, and provides information about the control of regulator proteins in functionally homogeneous gene subgroups.

We performed extensive comparison of TAFFEL to four other popular methods DAVID, GeneCodis, GSEA and FatiGO+, which are targeted to address similar challenges as TAFFEL. For full explanation of comparison results from forskolin dataset and methodology see Additional file 1 and for result tables from sIA dataset see Additional file 2.

Similar ways of clustering gene sets are implemented in GENERATOR [3], GOToolBox [6] and DAVID [4] tools. Our comparison between TAFFEL and DAVID clustering and standard EA indicates the advantage of clustering methods over standard EA: clustering can ease the interpretation of results by reducing the amount of resulting categories and may additionally highlight some potentially important categories not revealed as significant in the whole gene set. Furthermore, IEA implemented in TAFFEL presents two new improvements. First, pointing a few clusters out of many eases the interpretation of results. Secondly, TAFFEL IEA can point gene clusters or GO terms that are not statistically significantly enriched in the whole gene list, and thus not reported by standard EA or DAVID clustering, but are still potentially biologically meaningful due to enrichment of TF annotations.

FatiGO+ [5] tool provides information about the enriched GO terms and TFs using TRANSFAC and cisRED databases, similarly as TAFFEL. The main difference between FatiGO+ and TAFFEL is that FatiGO+ searches enrichment in the complete list of DE genes and does not consider genes as subsets like TAFFEL. Also, it does not provide relation between TFs and different enriched biological processes. The same results can be obtained from TAFFEL GO and TF tree root levels, which analyze the enrichment in the complete gene set. Additionally, TAFFEL clustering and IEA can discover novel themes from the data and provide clues to the regulatory control of identified biological processes.

Analysis with GSEA [11] method did not produce very good results with the tested data sets. No annotations were significant after multiple testing corrections. The problems regarding the robustness of GSEA with various situations have been reported before [27,28]. However the strength of the GSEA method is that the analyst does not need to define fixed statistical cut-off for producing differentially expressed gene set. GSEA seeks the enrichment of terms (functional classifications, transcription factor binding sites, etc.) in the top or in the tail of the gene set, which is sorted according to e.g. fold change or p-values. Nevertheless GSEA seeks the enrichment of annotations separately and does not consider any relations between annotation terms as TAFFEL does.

We considered the comparison of TAFFEL to GeneCodis as the most important of all presented comparisons as these two tools address partly the same concern by seeking relations between different annotation systems within a set of genes. GeneCodis, however, does not perform any clustering and therefore may miss important biological phenomena, which are not enriched in the whole gene set but in a subset of genes. It should be noted that GeneCodis does not particularly aim at finding only relations between GO terms and TFs, but rather co-occurrences of any terms within one or several annotation systems. This can be important clustering in itself, and is provided also by TAFFEL in the form of enriched GO-terms or TFs in each resulting gene cluster. However, the results from GeneCodis for our data sets show a very large list of annotations with ambiguous repetition of the same GO terms and/or TFs coupling with each other in multiple various combinations (see additional file 2 for the 50 first ranks of the 4538 total ranks reported significant after FDR correction). The numbers of genes associated to such co-occurring annotations were also very low although reported significant. Using the forskolin data set, the maximum of associated genes was 6 with co-occurring annotations including terms from only one annotation system and 4 with co-occurring annotations including terms from both GO and TF annotation systems. In order to compare GeneCodis to TAFFEL IEA method, we paid special attention to the few co-occurrences including both GO terms and TFs (see Additional file 1 for forskolin dataset and Additional file 2 for full results from both datasets). Some of the themes such as transcription regulation were common with the results from other tools. However, the results contained ambiguous repetition of the same process with several different sets of TFs. As a comparison, TAFFEL clusters resulting from IEA in Table 1 include 22 - 58 genes and of these genes the independently enriched (statistically significant after FDR correction) GO or TF terms cover 20 - 60%. This suggests that it would be advantageous to perform such clustering analysis instead of associating individual GO terms and TF annotations. GeneCodis may however work better when dealing with two or more annotation systems with highly overlapping annotations, such as GO and KEGG.

## Discussion

We present a novel method for the analysis of differentially expressed (DE) genes for the discovery of co-functional and co-regulated subsets of genes, and for further analysis of such clusters with functional annotations and regulatory protein information. As information about gene regulatory elements, we have used TF predictions and annotations from cisRED database where putative binding sites are validated in terms of evolutionary conservation [13]. Such validation has shown to be advantageous as it can significantly reduce the amount of false positives in predictions [29,30]. Moreover, our clustering of genes using TF information as well as the further validation of discovered clusters using functional annotations should reveal relevant patterns from the data and reduce amount of noise.

A major limitation in our and many other methods employing GO and TF data is that the knowledge on gene functions (the GO annotations) [31] and regulation (TFs) is incomplete. Furthermore the GO annotations are biased towards well-studied biological phenomena and the predicted TF binding sites (cisRED) often contain large number of false positives [32]. Still the clustering method alleviates this problem in the sense that the clustering is not driven by randomly distributed annotations (false positives or negatives) but by stable annotations shared by many genes. The constantly improving quality of the annotations is also likely to improve the results obtained using our method. It should also be noted that gene expression is not necessarily functional in the sense that co-expressed or similarly expressed genes do not necessarily share any GO annotations. Thus our clustering approach does not necessarily produce clusters of co-expressed genes which likely results to fewer significant IEA clusters. Also the used AIC method for cluster number selection is not necessarily optimal, but rather it strikes a good balance between accuracy and number of parameters. The cluster number selection is a very general problem and usually there is no single best solution for every dataset (see for example reviews [33,34]). In our method we use cluster number selection as a guide for the analyst to focus on some particular clustering level to start the analysis with.

The result of TAFFEL analysis for the DE genes after forskolin treatment of human HEK293T cells in culture showed expected results at the first level of clustering tree, e.g., the enrichment of cAMP related GO terms and CREB TF. Interestingly, the clustering analysis was able to identify a piece of a complex MAPK-AP1-AhR related transcription network, related to proliferation and regulation of metabolism. The most prominent result was the independent enrichment of AhR and HES-1 TFs in the macromolecule localization related gene cluster. The AhR activation alone causes up-regulation of xenobiotic-metabolizing enzymes. MAP kinases are known to be involved in process in which AhR and the heat-shock chaperone complex are translocated to nucleus [35]. AhR is also phosphorylated by calcium-controlled protein kinase C, and by several other kinases [35]. JUN and ELK1, included in the cluster, are not typically considered as direct target for the AhR agonist, but are known to be phosphorylated after AhR activation [36]. However, although the enrichment of AhR TF was not the most expected result, recent information shows that cAMP is indeed a direct mediator of AhR signaling [37]. Hairy and Enhancer of Split homolog-1 (HES-1) is a transcriptional repressor with basic helix-loop-helix structure. It has been suggested that HES-1 and AhR factors have cross-talk [38,39] although the cross-talk between AhR and other transcription factors is complex and poorly understood [35]. Recent literature indicates that AhR has a role in regulation of the transcription in human HEK293T cells and in mouse kidney (Boutros et al., 2009), generally agreeing with results of TAFFEL analysis.

In the analysis of over and under-expressed genes in the ruptured saccular intracranial aneurysm (sIA) walls TAFFEL identified several interesting clusters, some in line with prior data [15-18], some providing basis for new hypotheses on mechanism behind the sIA wall rupture. In the TAFFEL analysis for the over-expressed genes, previously described phenomena of MAPK and apoptotic signalling related to the sIA wall rupture [18] were detected. MAPK is also known to control a wide spectrum of other biological processes, including the cell cycle, cellular metabolism, motility and survival. However, the anti-apoptotic and pro-apoptotic control of MAPKS is not presently well known [40]. Secondly, TAFFEL results for the over-expressed genes support forming new hypotheses relating to the signalling that ensures endothelial integrity. Inflammatory cell infiltration has been reported to associate to the sIA wall rupture [15] but the etiology or mechanisms for this phenomenon remain unknown. Our data suggests the possibility that abnormal function of Tal-1 transcription factor, being in the centre of endothelial cell integrity preserving regulatory cascade of TGF-β and VE-cadherin signalling, might lead to excess vascular permeability and endothelial dysfunction, leading in turn to enhanced inflammatory cell infiltration and vascular wall instability.

Other significant IEA cluster for the under-expressed genes in the ruptured sIA walls was the regulation of oxidation reduction and metabolism genes by NF1C. NF1C activity is repressed by oxidative stress [23] and thus the down-regulation of the genes in this cluster might be caused by inactivation of NF1C by oxidative stress possibly present in the ruptured aneurysms [19]. The exact consequences of down-regulation of these metabolic genes in ruptured aneurysms must be investigated in further studies.

Final observation from IEA for the under-expressed genes in the ruptured sIA walls was the regulation of metallothioneins (MT; genes associated to GO term *metal ion transport*) by MTF-1 transcription factor. MT activation and reduced zinc bioavailability is known to associate with aging and cardiovascular diseases in the elderly [22]. It is also known that the risk of sIA wall rupture and subarachnoid hemorrhage increases with age [41]. Although MTF-1 is mainly vascular protective, chronic low grade inflammation can maintain long-term elevation of MTs, which in turn may lead to pro-inflammatory response plausibly due to decreased zinc bioavailability [22]. Thus, the active regulation of MT genes by MTF-1, proposed by TAFFEL, suggests that long-term inflammation and zinc deficiency may play crucial roles in the rupture, caused by either a de-stabilization or reactive changes in the sIA wall tissue. Dysregulation of other metal ions such as calcium might be other outcome of MTF-1 signalling. In fact, a calcium channel blocker nimodipine is recommended as a standard treatment for patients with aneurysmal subarachnoid hemorrhage to prevent secondary vasospasm and ischemic brain injury [42].

## Conclusions

In conclusion, we have demonstrated that the developed method and TAFFEL tool give new insight in to the analysis of differentially expressed genes and can generate novel hypotheses. Our comparison to other popular methods showed that the IEA method implemented in TAFFEL can find important biological phenomena, which are not reported by other methods at all.

Firstly, the analysis of forskolin-treated HEK293T cells indicates that TAFFEL will identify well-known and expected phenomena such as differential expression of CREB regulated genes, but can also lead to new hypotheses, e.g., on the role of AhR. Secondly, the results with the sIA wall rupture related data give confidence to the usefulness of TAFFEL in the analysis of complex and poorly characterized clinical conditions, affected by inherited and acquired risk factors. These findings suggest that TAFFEL is an efficient method to generate new hypotheses to be further tested in basic or applied molecular genetic research. The testing of such hypotheses is crucial for finding novel targets for new biological approaches, e.g, diagnostic tests for the identification of sIA carriers in population, or non-invasive methods to close or stabilize the rupture-prone sIA wall.

## Methods

### Annotation data sources

For the functional grouping of genes, TAFFEL uses Gene Ontology [1] annotations (December 2008 release used in this study) from Ensembl database [43] (version 53 used in this study). The included species are human, mouse, rat and *C. elegans*. The current version of TAFFEL can use biological process and molecular function ontologies from GO, either separately or in parallel.

Secondly, TAFFEL uses information about predicted TFBSs available in the public cisRED database [13], containing genome wide collections of sequence motifs conserved in gene regulatory regions. The motifs have been annotated by transcription factors (TFs) found in TRANSFAC [44] and JASPAR [45] databases. In TAFFEL, we have included all TF annotations from both of these databases that have similarity p-value < 0.001 with the found sequence motif. We have included data for human (version 9), mouse (version 4) and *C. elegans *(version 4).

### Gene clustering method

In order to perform gene clustering, associations between genes and annotations (GO terms and TFs) are represented as a binary matrix. Each row in the matrix represents a gene and each column represents an annotation. In the matrix, the cell value one indicates association and zero indicates no association between the row (gene) and the column (annotation) (Figure 1). For clustering, we apply Non-negative matrix factorization (NMF) [46] based approach. This approach has been advantageous in clustering of sparse binary data and finding clusters that are defined in a (possibly small) subset of all data attributes [47]. Both of these features are important in our cases described here. Firstly, the data are sparse by nature. Secondly, one set of genes often associates to numerous biological attributes (TFs and GO terms), many of which may not be relevant [3].

### Selection of number of clusters

In order to choose a clustering solution with suitable balance between goodness of fit in the data and complexity, TAFFEL uses Akaike Information Criterion (AIC) [48] for statistical model selection. AIC is calculated by taking the number of parameters of the statistical model representing the evaluated clustering solution and subtracting them from the maximized log-likelihood of the data for the same model. Due to simplicity and robustness of the method, it has been widely used in similar clustering applications (see e.g. [49-51]).

As the abundance of dimensions (GO terms or TFs) in the gene annotation data are distributed randomly in resulting clusters, the clusters tend to exist in a relatively small subset of all dimensions [3]. Besides being problematic for clustering, this behaviour is also problematic for model selection. The model selection tends to be overwhelmed by such dimension and systematically favour a result with only one or a few clusters with different data sets. Thus, we also calculated a modified AIC, referred to as dAIC, for which we used only the dimensions that are distributed in a non-random fashion in at least one of the clustering solutions with >2 clusters in the whole TAFFEL tree. This was tested by comparing the AIC score of the dimension in the whole gene list versus the AIC score in each clustering solution. If the AIC score is better (smaller) in any of the clustering solutions, then the evaluated dimension was included in calculus of dAIC. The same set of dimensions was then used for calculating dAIC for different clustering solutions including the whole gene list as one cluster. This feature selection filtered out at least 50% of the GO terms in our forskolin and sIA datasets (see *Results *section for detailed description of the datasets). When the remaining dimensions were used for calculating AIC score the number of selected clusters was systematically higher than when using all dimensions.

### Cluster statistics

The statistical testing of enrichment in TAFFEL is calculated using Fisher's exact test. Only annotations with occurrences in a cluster are used in the testing. The resulting p-values are corrected for multiple testing using Benjamini-Hochberg False Discovery Rate (FDR) [52].

The interpretation of p-values reported by TAFFEL warrants a special note. In each cluster, enrichment is analyzed for the annotations of the same (Dependent Enrichment Analysis, DEA) and different (IEA) annotation system that was used in clustering. The p-values resulting from IEA have reasonable statistical interpretation as they test null hypotheses such as: "*TF x is not dependent of the gene group y homogeneous in GO terms*". Due to statistical independence between variables *x *and *y*, these p-values can be used reasonably to detect their biological significance and dependence of each cluster. As an opposite, the p-values from DEA would test null hypotheses such as: "*GO term x is not dependent of gene group y homogeneous in GO terms*". Here, variable *y *is statistically dependent on *x *and thus treating the resulting values as standard p-values for statistical decision-making would lead to circular argumentation. Still, these values from DEA are suitable as relative enrichment scores representing the most characteristic annotations in each cluster.

The inter-correlation measurements are also calculated using Fisher's exact test with Benjamini-Hochberg correction. As dependencies exist among the clusters between different inter-correlation comparisons, the correction tends to be highly conservative for this situation and should be interpreted with care.

The correlation between each cluster pair between the adjacent clustering solutions in the same clustering tree is calculated using standard correlation between two binomial distributions representing the gene memberships in the clusters.

### Processing of demonstration microarray data sets

Gene expression microarray data (GSE2060 Affymetrix Human Genome U133A Array) concerning the effect of forskolin in human HEK293T culture was downloaded from Gene Expression Omnibus (GEO) and normalized using RMA method. Forskolin-treated and control HEK293T cells (both in duplicates) in culture were compared at 4 hours to find out differentially expressed genes. Welch's t-test with Benjamini-Hochberg correction was used. Due to a low number of replicates, the fold change was used as an additional measure for filtering. P-value < 0.05 and fold change > 1.25 resulted in 691 differentially expressed genes.

Whole genome expression data of 11 ruptured and 8 unruptured sIA wall samples resected after microsurgical clipping of the sIA neck were compared using Affymetrix HG-U133 Plus 2.0 microarrays [19]. The data was RMA normalized and compared using Welch's t-test with Benjamini-Hochberg correction for p-values. Genes with p-value < 0.05 were regarded as differentially expressed genes. This resulted in 498 overexpressed and 491 underexpressed genes in the ruptured sIA wall group.

## Abbreviations

DE: differentially expressed; EA: enrichment analysis; IEA: independent enrichment analysis; FDR: False Discovery Rate; sIA: saccular intracranial aneurysm; cAMP: a cyclic AMP; GO: Gene Ontology; TF: transcription factor; TFBS: transcription factor binding site; KEGG: Kyoto Encyclopedia of Genes and Genomes; GEO: Gene Expression Omnibus; RMA: Robust Multi-array Average; CREB: Cyclic AMP response element-binding; GSEA: Gene Set Enrichment Analysis; AIC: Akaike Information Criterion

## Authors' contributions

PP and MK presented the idea and co-developed the method, PP coordinated the research, GW co-coordinated the research. MK designed and implemented the program and performed analysis and interpretation of sIA related data sets, PP did additional programming. MS analyzed the forskolin treated cell culture data set. JP helped in the development of software and writing the manuscript. SYH, JJ and MF provided the sIA data and helped in interpretation of analysis results. PP and MK wrote the draft of the manuscript, which was refined by MK, JP, MS, GW, MF and JJ.

All authors read and approved the final manuscript.

## Supplementary Material

Additional file 1 **Detailed method comparison methodology and interpretation**. Explanation of methods used and detailed interpretation of results when comparing TAFFEL to other methods. Click here for file

Additional file 2 **Full comparative analysis results**. Full results obtained for both forskolin and aneurysm datasets using different methods. Click here for file
