# Supplementary material for: TAFFEL: Independent Enrichment Analysis of gene sets
Source: BMC Bioinformatics. 2011 May 19;12:171. doi: 10.1186/1471-2105-12-171 (PMC3120704; doi:10.1186/1471-2105-12-171)
Supplement: Additional file 1 — Detailed method comparison methodology and interpretation. Explanation of methods used and detailed interpretation of results when comparing TAFFEL to other methods. [file 1471-2105-12-171-S1.DOC]

### TAFFEL method comparison

First we compared TAFFEL results to results from DAVID Functional Annotation Clustering tool with default parameters [1]. The major difference in functionality between these tools is that whereas TAFFEL clusters genes using GO-term memberships as data. DAVID clusters GO-terms using gene memberships as data. In DAVID the clustering is also performed using fuzzy clustering algorithm with kappa-statistics as a similarity measure. However both methods aim partly at similar type of result including a reduced number of reported classes (i.e. sets of GO-terms) for the user. We mapped the most enriched GO-term from each cluster from single TAFFEL GO-tree level (pointed by dAIC measure) and DAVID clustering result against a standard sorted list of enriched GO-terms to see similarities and differences in the results reported (see Table 1 for results for forskolin dataset and Additional file 2 for results for sIA datasets). When inspecting results only in the light of GO terms the largest difference between DAVID and TAFFEL is the tendency of TAFFEL to produce fewer clusters than DAVID. Both clustering methods highlight themes that are not on the top in the sorted list such as *regulation of transcription* and related GO terms in forskolin treated cell culture dataset. However biological meaningfulness of these themes is questionable because they are statistically insignificant and DAVID clustering method provides no further information. In turn TAFFEL IEA points out statistically significant themes such as *transcription from RNA polymerase II* related gene cluster driven by AhR and HES-1 but also GO-terms that are statistically insignificant in standard sorted list and DAVID such as *macromolecule localization* related gene cluster independently enriching FOXO1 TF in dataset for forskolin treated cell culture (Table 1). Both of these GO terms are also present in the complete DAVID result report but the latter term may not seem important due to statistical insignificance. Comparison of TAFFEL to standard EA and DAVID using data set from ruptured and unruptured sIA walls shows also interesting points as hypotheses about MTF-1 driven metal ion transport in up regulated genes and NF1C driven oxidation reduction in down regulated genes (see Manuscript Table 1 for TAFFEL result and Additional file 2 for DAVID results) are only pointed out by the IEA implemented in TAFFEL.

Next we performed a comparison between TAFFEL and FatiGO+ [2] a tool that applies Fisher’s Exact test to seek over-represented annotations among several different annotation systems. In order to compare FatiGO+ against TAFFEL we used it to analyze the enrichment of TFs from TRANSFAC database mapped to gene regulatory regions via cisRED and biological process terms from GO. FatiGO+ results for DE genes in forskolin treated cells show the enrichment of cell cycle and death related terms and E2F TF, which is also reported by TAFFEL IEA method (also enriched in the root of TAFFEL TF tree representing the standard way of enrichment analysis) (Table 2).

Next a comparison between TAFFEL and GeneCodis [3] tool was performed using the same forskolin dataset. GeneCodis aims at discovery of sets of annotations from the same or different annotation systems that co-occur in the given set of genes. This can be used partly similarly as TAFFEL to find associations between TFs and GO-terms that are enriched in the input set of genes. GeneCodis co-occurrence analysis results in 4538 individual or combined classes of GO biological process terms and TFs after FDR correction. For simplicity and because the lower ranks are mostly repetition, only the 50 highest-ranking classes are considered here. Table 4 shows the classes containing combination of both TF and GO terms as those are of particular interest when comparing GeneCodis to TAFFEL. As the most notable result GeneCodis has linked transcription regulation process in several result classes to various sets of transcription factors including FOXO4, NFAT, PAX3 and FREAC2. On the other hand transcription regulation theme has been linked to signal transduction in class with rank 37 including also TFs FOXO4 and FREAC2. In addition signal transduction has been linked alone to STAT5B and LEF1 in another class (rank 35). As shown in the table 4 the terms signal transduction transcription regulation FOXO. FREAC and PAX3 are also visible in TAFFEL results. Both methods link the signal transduction theme with FOXO TFs. Otherwise the linking between TFs and GO terms is quite different even among the terms produced by both methods. The themes reported by TAFFEL IEA and not reported by GeneCodis GO versus TF term combinations are relations between E2F-4 and organelle organization and role of AhR and HES-1 in transcription regulation. In turn the linking between DNA damage correction and MEIS1 and MAZ TFs were not reported by TAFFEL or other compared methods.

Finally a comparison between TAFFEL and GSEA [4] was performed. GSEA performs analysis for a ranked gene list using *ad hoc* modification of Kolmogorov-Smirnov statistics. In GSEA results DE genes from forskolin treated cells no annotations were significant after multiple testing correction. Observations are therefore based on significant nominal p-values. We observed terms with significant positive enrichment score only (ES; table 4). The results show quite a low number of significant GO or TF terms. The similarity between the GSEA results with results from TAFFEL or other tools is also very low. Only few themes were same as in TAFFEL results including signalling related GO-term and TFs HNF4. FREAC and CHX10.

### Methods

Method comparison was performed using all three different gene sets: DE genes from forskolin treated cell culture and up and down regulated genes from comparison of ruptured vs. unruptured sIA walls. As a statistical background gene set for each enrichment analysis tool the complete genome was used. For comparison the best scoring TAFFEL clustering solution (a level of tree) was chosen using dAIC measure from GO tree and TF tree.

In comparison of TAFFEL against DAVID the most enriched GO term from each cluster was mapped against the standard EA result list of sorted GO terms (Table 1 for forskolin dataset and Additional file 1 for plain results from sIA datasets). The standard sorted EA list was obtained from the TAFFEL root level although other EA tools such as Functional Annotation Chart tool in DAVID could produce similar list. The term list included all biological process terms that associated to the analyzed gene list and that had <1000 genes associated in the complete genome (default TAFFEL threshold to exclude very general GO-terms). Similar mapping was produced for DAVID [1] clusters obtained with default clustering parameters. Clusters were sorted in output according to the cluster enrichment scores (geometric mean of p-values of member GO-terms [1]). Only DAVID clusters which had the GO term enrichment p-value < 0.05 (Benjamini-Hochberg corrected) assigned with the most enriched GO-term were included to the comparison. For comparison purposes also the cluster and term ranks of GO terms were also reported. Rows of standard EA output that did not map to any TAFFEL or DAVID clusters were deleted.

Comparison of TAFFEL against FatiGO+, GSEA and GeneCodis was performed by obtaining all biological process GO-terms and transcription factors that were reported significant by each method and mapping the TAFFEL clusters from the dAIC selected TF and GO tree levels against these results. In each TAFFEL cluster the rank of the GO term or TF that was same with or similar to the each reported term from other compared tools was recorded. As a cluster that maps the best between TAFFEL and a reported term from each compared tool the TAFFEL cluster with the highest rank for that term was chosen (Tables 2, 3 and 4 for forskolin dataset and Additional file 1 for plain results from sIA dataset).

For FatiGO+ the GO-term biological process annotations and TRANSFAC transcription factor mappings against 5kb flanking regions of genes were tested for over-representation (one sided test) by using Benjamini-Hochberg FDR correction for multiple testing.

With GeneCodis2 the default settings were used including Fisher’s exact test for statistical testing. Benjamini-Hochberg FDR correction for multiple testing and three as minimum number of associated genes for reported terms. The tool uses TF mappings obtained from GSEA dataset [4]. Only co-occurring annotation sets including both GO-term and TF were reported.

In GSEA results, none of the multiple testing corrected p-values were statistically significant and to be able to somehow practically compare TAFFEL and GSEA methodologies, annotations with nominal p <0.05 were reported.

**Table 1.** Comparison of DAVID and TAFFEL tools for DE genes from comparison of forskolin treated cell culture and control. Results from the two tools are also mapped against results from standard EA. The columns from left to right: rank and GO term from sorted list of GO terms resulting from standard EA; Cluster and term rank within cluster for TAFFEL and DAVID result clusters; For TAFFEL. The clusters resulting from IEA and the corresponding independently enriched TFs are indicated.

**Table 2.** Results from FatiGO+ with comparison to TAFFEL for DE genes from comparison of forskolin treated cell culture and control. The columns from left to right: term and its FDR corrected p-value resulting from FatiGO+ analysis. Cluster rank and the term rank in the cluster from TAFFEL GO and TF trees with indication of enriched or independently enriched terms. * indicates that E2F4 was observed in TAFFEL results instead of E2F1. Nominal indicates nominal significance (p<0.05 in uncorrected p-value).

**Table 3.** Results from GeneCodis with comparison to TAFFEL for DE genes from comparison of forskolin treated cell culture and control. The columns from left to right: rank. FDR corrected p-value and co-occurring set of terms resulting from GeneCodis analysis (only set of terms containing both GO-terms and TFs are presented); Cluster rank and the term rank in the cluster from TAFFEL GO and TF trees with indication of enriched or independently enriched terms. Total amount of co-occurring term sets reported as significant (FDR corrected p-value<0.05) was 4538. Only 50 first ranks were considered here due to simplicity. * indicates that GO-terms from same branch were enriched among top five terms. ** indicates that FOXO1 was observed in TAFFEL results instead of FOXO4. *** indicates that FREAC4 was observed in TAFFEL results instead of FREAC2.

**Table 4.** Results from GSEA with comparison to TAFFEL for DE genes from comparison of forskolin treated cell culture and control. The columns from left to right: GO term or TF (term). Number of associated genes (N). Nominal p-value (NOM P) and FDR corrected q-value (FDR Q) resulting from GSEA analysis; Cluster rank (Cluster) and the term rank (Rank) in the cluster from TAFFEL GO and TF trees with indication of enriched or independently enriched terms (IEA/EA). * indicates that GO-terms from same branch were enriched among top five terms. ** indicates that FREAC4 was observed in TAFFEL results instead of FREAC2.

**Table 1.**

| **STANDARD EA** | |  |  | **TAFFEL** | | | **DAVID** | |
| --- | --- | --- | --- | --- | --- | --- | --- | --- |
| Rank | GO-term | P | N | Cluster | Rank | IEA | Cluster | Rank |
| 1 | cell cycle | 1.42E-06 | 65 | 5 | 1 |  | 5 | 1 |
| 7 | cell death | 6.55E-05 | 61 | 3 | 1 |  | 4 | 5 |
| 12 | negative regulation of metabolic process | 1.95E-04 | 40 | 2 | 7 |  | 1 | 3 |
| 17 | cellular macromolecular complex assembly | 3.02E-04 | 40 | 4 | 1 |  | - | - |
| 18 | regulation of transcription from RNA polymerase II promoter | 3.27E-04 | 39 | 6 | >10 | HES-1; AhR | 6 | 1 |
| 22 | nucleosome assembly | 4.47E-04 | 19 | 4 | 9 |  | 3 | 1 |
| 31 | transcription from RNA polymerase II promoter | 8.24E-04 | 46 | 6 | 1 | HES-1; AhR | 6 | 2 |
| 52 | negative regulation of macromolecule biosynthetic process | 5.00E-03 | 28 | 2 | 1 |  | - | - |
| 56 | DNA metabolic process | 7.00E-03 | 35 | 5 | 7 |  | 7 | 1 |
| 90 | regulation of organelle organization and biogenesis | 0.04 | 11 | 10 | 1 |  | 57 | >10 |
| 128 | cell morphogenesis | 0.06 | 24 | 8 | 9 |  | 10 | 1 |
| 151 | lipid metabolic process | 0.07 | 41 | 7 | 1 |  | - | - |
| 162 | macromolecule localization | 0.08 | 49 | 1 | 1 | FOXO1 | 16 | 2 |
| 201 | nervous system development | 0.11 | 42 | 8 | 1 |  | - | - |
| 890 | nucleobase. nucleoside. nucleotide and nucleic acid catabolic process | 0.19 | 1 | - | - |  | 9 | 1 |
| 1256 | cell adhesion | 0.31 | 33 | 11 | 1 |  | 20 | 3 |

Table 2.

| **FatiGO+** |  | **TAFFEL GO TREE** | | | **TAFFEL TF TREE** | | |
| --- | --- | --- | --- | --- | --- | --- | --- |
| Term | P | Cluster | Rank | IEA/EA | Cluster | Rank | IEA/EA |
| cell cycle | 8.55E-06 | 5 | 1 | NF-Y (nominal) |  |  |  |
| death | 2.30E-03 | 3 | 2 |  |  |  |  |
| cell cycle process | 1.69E-03 | 5 | 3 |  |  |  |  |
| mitotic cell cycle | 2.50E-02 | 5 | 2 | NF-Y (nominal) |  |  |  |
| organelle organization and biogenesis | 2.50E-02 | 10 | 1 |  | 1 | 1 | E2F-4 |
| regulation of cell cycle | 1.12E-03 | 5 | >10 |  |  |  |  |
| DNA metabolic process | 2.38E-02 | 5 | 7 |  |  |  |  |
| negative regulation of metabolic process | 2.45E-02 | 5 | >10 |  |  |  |  |
| cell cycle phase | 3.58E-02 | 5 | 4 |  |  |  |  |
| E2F-1 | 1.15E-11 |  |  |  | 1 | 1* | Organelle organization |
| E2F-1:DP-1 | 1.14E-04 |  |  |  | 1 | 1* | Organelle organization |
| E2F | 2.67E-04 |  |  |  | 1 | 1* | Organelle organization |
| E2F-1:DP-2 | 2.67E-04 |  |  |  | 1 | 1* | Organelle organization |
| E2F-4:DP-2 | 2.67E-04 |  |  |  | 1 | 1 | Organelle organization |

Table 3.

| **GENECODIS** | |  | **TAFFEL GO-TREE** | |  | **TAFFEL TF-TREE** | |
| --- | --- | --- | --- | --- | --- | --- | --- |
| Rank | P | Term | Cluster | Rank | IEA/EA | Cluster | Rank |
| 10 | 1.4E-03 | MEIS1_01 |  |  |  |  |  |
|  |  | MAZ_Q6 |  |  |  |  |  |
|  |  | response to DNA damage stimulus |  |  |  |  |  |
| 12 | 3.1E-03 | regulation of transcription. DNA-dependent | 6 | * | AhR & HES-1 |  |  |
|  |  | FOXO4_01 | 1 | 1** | macromolecule localization |  |  |
|  |  | NFAT_Q4_01 |  |  |  |  |  |
|  |  | PAX3_B | 11 | 1 | Pax-5 (nominal) | 13 | 1 |
| 14 | 3.2E-03 | regulation of transcription. DNA-dependent | 6 | * | AhR & HES-1 |  |  |
|  |  | NFAT_Q4_01 |  |  |  |  |  |
|  |  | PAX3_B |  |  |  | 13 | 1 |
| 27 | 3.8E-03 | FOXO4_01 | 1 | 1** | macromolecule localization |  |  |
|  |  | MAZ_Q6 |  |  |  |  |  |
|  |  | LEF1_Q2 |  |  |  |  |  |
|  |  | multicellular organismal development |  |  |  |  |  |
|  |  | SOX5_01 |  |  |  |  |  |
| 29 | 4.2E-03 | regulation of transcription. DNA-dependent | 6 | * | AhR & HES-1 |  |  |
|  |  | FOXO4_01 | 1 | 1** | macromolecule localization/GTP signal transduction |  |  |
|  |  | signal transduction | 1 | * | FOXO1 |  |  |
| 35 | 5.0E-03 | STAT5B_01 |  |  |  |  |  |
|  |  | signal transduction | 1 | * | FOXO1 |  |  |
|  |  | LEF1_Q2 |  |  |  |  |  |
| 37 | 5.0E-03 | regulation of transcription. DNA-dependent | 6 | * | AhR & HES-1 |  |  |
|  |  | FOXO4_01 | 1 | 1** | macromolecule localization/GTP signal transduction |  |  |
|  |  | signal transduction | 1 | * | FOXO1 |  |  |
|  |  | FREAC2_01 | 2 | 1*** | Negative regulation of macromolecule biosyntetic process |  |  |

Table 4.

| **GSEA** |  |  |  | **TAFFEL GO TREE** | | | **TAFFEL TF TREE** | | |
| --- | --- | --- | --- | --- | --- | --- | --- | --- | --- |
| **TERM** | **N** | **NOM P** | **FDR Q** | **Cluster** | **Rank** | **IEA/EA** | **Cluster** | **Rank** | **IEA/EA** |
| intracellular signaling cascade | 28 | 0.03 | 1 | 1 | * | FOXO1 |  |  |  |
| cell fraction | 24 | 0.02 | 0.98 |  |  |  |  |  |  |
| V$HSF2_01 | 18 | 0.00 | 0.12 |  |  |  |  |  |  |
| V$HSF1_01 | 5 | 0.01 | 0.26 |  |  |  |  |  |  |
| V$FREAC2_01 | 1 | 0.01 | 0.21 | 2 | 1** | Negative regulation of macromolecule biosynthetic process | | | |
| V$HNF4_01_B | 7 | 0.03 | 0.40 | 8 | 2 | nervous system development | | | |
| CTGCAGY UNKNOWN | 4 | 0.02 | 0.33 |  |  |  |  |  |  |
| V$WHN_B | 6 | 0.03 | 0.29 |  |  |  |  |  |  |
| V$CHX10_01 | 7 | 0.04 | 0.31 |  |  |  | 3 | 2 | covalent chromatin modification |
| V$HSF1_01 | 5 | 0.05 | 0.29 |  |  |  |  |  |  |

References

1. Dennis G,Jr, Sherman BT, Hosack DA, Yang J, Gao W, Lane HC, Lempicki RA: **DAVID: Database for Annotation, Visualization, and Integrated Discovery.** Genome Biol 2003, **4**(5):P3.

2. Al-Shahrour F, Diaz-Uriarte R, Dopazo J: **FatiGO: a web tool for finding significant associations of Gene Ontology terms with groups of genes.** Bioinformatics 2004, **20**(4):578-580.

3. Carmona-Saez P, Chagoyen M, Tirado F, Carazo JM, Pascual-Montano A: **GENECODIS: a web-based tool for finding significant concurrent annotations in gene lists.** Genome Biol 2007, **8**(1):R3.

4. Subramanian A, Tamayo P, Mootha VK, Mukherjee S, Ebert BL, Gillette MA, Paulovich A, Pomeroy SL, Golub TR, Lander ES, Mesirov JP: **Gene set enrichment analysis: a knowledge-based approach for interpreting genome-wide expression profiles.** Proc Natl Acad Sci U S A 2005, **102**(43):15545-15550.
